# Comparing the uplink performance of 3D and 2D antenna models in THz networks in the presence of joint human and wall blockages

**DOI:** 10.1371/journal.pone.0351223

**Published:** 2026-06-12

**Authors:** Tahniyat Aslam, Irfan Ahmed, Sundus Ali

**Affiliations:** 1 College of Computer Science & Information Systems, Institute of Business Management, Karachi, Pakistan; 2 Department of Telecommunications Engineering, NED University of Engineering and Technology, Karachi, Pakistan; Universite Cote d’Azur, FRANCE

## Abstract

Terahertz (THz) communication is considered as a key technology enabler for realizing Sixth Generation (6G) network. THz band communication offers several promising advantages, but numerous challenges are expected due to the inherent limitations of propagation at THz frequencies in the 6G network, such as path loss, interference, human and wall blockages, etc. In retrospect, THz band communication finds its use in indoor network deployments. In this paper, a framework is developed to analyze the impact of the uplink performance of a single-tier THz network, incorporating the impact of wall and human blockages in the indoor environment. To model a practical system, 3D antenna model have been employed, which accounts for both horizontal and vertical radiation patterns, whose performance have been benchmarked against 2D antenna model that accounts only for horizontal direction. This evaluation has enabled us to highlight the impact of practical antenna models on THz communication performance. Using the developed system model, generalized expressions for uplink mean interference, uplink coverage probability, and area spectral efficiency have been derived. The impact of THz uplink network performance has been analyzed using an antenna model with varying user equipment heights and different main lobe beam widths, as well as considering different path loss exponents for Line-of-Sight (LOS) and Non-Line-of-Sight (NLOS) conditions. The analytical results obtained against different network conditions have been compared and validated against Monte Carlo simulations and both have been found in agreement.

## 1. Introduction

The Terahertz (THz) frequency bands (0.1–10 THz) in wireless communication are considered as the key enablers of future Sixth generation (6G) cellular networks, in particular, to enable Enhanced Mobile Broadband Plus (eMBBPlus) networks [1–5]. The THz band has the potential to employ a wider bandwidth of the order of tens of GBs [6–8], which is an essential technology for 6G wireless systems. THz frequencies offer numerous advantages [9–12], such as sufficient spectrum, dense deployment, and massive machines and devices connection opportunities. Some of such applications include, but are not limited to, remote surgery and holographic communication, etc. Applications like remote surgery are envisioned to transform healthcare services. The above-mentioned applications necessitate a higher data rate (up to 1 Tbps) and lower latency rate (as low as 100 µs) to meet real-time communication requirements, which are targeted to be achieved using THz communication technology under the 6G standard. Moreover, holographic communication, which enables distant users to represent their presence, operates in 3D space with audio that can mimic various physical attributes and requires more bandwidth (up to 100 GHz), which is not supported by Fifth Generation (5G) technology enablers [13–16].

Regardless of several promising advantages of employing THz communication, there are several challenges documented that contribute to the inherent propagation limitations, such as path loss, blockages, interference, etc [17–22]. To fully exploit the advantages of the THz frequency band in an indoor environment, it is necessary to address the issues of blockages caused by the human body and walls. Similarly, the impact of path loss, interference, and the directional antenna model needs to be considered.

Blockages are more susceptible to THz signals. Particularly, moving humans and indoor obstructions (such as walls) can appear as impenetrable blockers. In [23,24], the authors discussed a blockage model named as Line-of-Sight (LOS) ball model for analyzing the THz frequency band. In [25], a Boolean model is presented for human body blockages, where humans are modeled as cylinders with their centers creating a Poisson Point Process (PPP). In [26], the double knife-edge (DKE) human blockage model is discussed, where a human blockage blocks the LOS link between the access point (AP) and the user within the specified area. The authors in [27], presented a comparison of different wall-generation methods and found that the Manhattan Poisson line process (MPLPs) is the most tractable method. In [28], the authors discussed the differentiation between the multiple rays propagation model and the corresponding indoor THz channel model, to express the link between the user and THz Access Point (TAP). A multi-ray propagation model comprises the LOS link and the number of Non-line-of-sight (NLOS) links reflected by the walls of a room [29,30], and it requires high computation complexity.

High directivity is an important feature in THz communication. The authors in [31], discussed the radiation patterns for the single cone model and cone-plus-sphere model to determine the antenna directivity. The author in [32], presented the simple flat-top antenna model, which improves tractability, but it fails to relate the effect of the directional pattern. The authors in [33], resolved the issue of the flat-top antenna model and proposed a Multi-Level Flat-Top (MLFT) antenna model, even though the narrow beams can ultimately lead to a noise-limited regime [34–36] in a hybrid network. The authors of [37], proposed a Multi-Cone (MC) model, which is estimated as cone-shaped beams that can increase the signal power and effectively improve the coverage. In 2D antenna models [31–37], the main lobes of TAPs and their associated User Equipment (UEs) are directed at each other only in the horizontal direction, ignoring the impact of vertical direction. The authors of [38], proposed a 3D Pyramidal-plus-Sphere sectored (PS) antenna model that accounts for both horizontal and vertical directions, and provides a more accurate representation of the complex propagation scenarios in downlink THz communication.

Several authors have presented the performance analysis of the single-tier THz network. The authors in [28], discussed a model in an indoor environment and derived the coverage probability for Tera wireless local-area network (WLAN) to study the impact of blockages caused by humans and wall blockages, whereas a simple directional antenna model has been employed to find tractable results. The authors of [39], proposed an uplink model for an indoor environment in the THz network, to investigate the co-channel interference and combined impact of phase noise while ignoring the impact of blockages caused by the wall and human body. In [40], a single-tier THz network has been modeled for investigating the characteristics of finite-sized downlinks such as directional antennas, human blockage, molecular absorption loss, etc, whereas the impact of NLOS propagation and wall blockage have not been considered.

Uplink performance analysis is particularly important in THz indoor scenarios because 6G follows a user-centric approach, and uplink traffic is expected to increase over time [41,42]. In uplink communication, the UE acts as the transmitter, and the link is fundamentally constrained by low transmit power and less precise beam alignment. These limitations become more critical at THz frequencies due to severe path loss, molecular absorption, and high susceptibility to human blockage especially when the UE is positioned at lower heights. In contrast, downlink performance typically shows better results due to higher transmit power and more robust beamforming capabilities at the base station.

Several authors have investigated the performance of single-tier THz communications [40,43], however, these works generally neglect multi-ray propagation effects. Moreover, existing literature rely on simplified 2D antenna models [31–37] to acquire tractable results. In addition, the modeling of blockages is also limited, as human blockages are often assumed to be static, and wall blockages are represented in a simplified manner [27,31]. Furthermore, most of the studies ignore the impact of NLOS PLE which may have significant impact on how the THz network behaves [40,43].

In this research, a system model for a single-tier THz network have been developed to investigate and analyze the performance of uplink THz communication, by employing a multi-ray propagation model, while incorporating the joint impact of human and wall blockages. For wall and human blockages, MPLP [28] and the DKE model [26], have been considered. This research presents a comparison of the oversimplified 2D antenna model with the realistic 3D antenna model [26,38] to analyze the impact of vertical height on the uplink coverage probability of THz communication. For a 2D antenna model, MC has been employed, which comprises a single main lobe and several side lobes that are directed at each other only in the horizontal direction. Whereas, in a 3D antenna model, PS has been used to analyze the 3D beams at both UEs and TAPs. The pyramid zone and sphere represent the main and side lobes’ beam widths of the antenna.

Furthermore, a comparative analysis of 3D antenna gains is conducted by considering different heights of UEs [44,45], specifically at the desk level and near the head level, to evaluate their impact on coverage probability under the blockages conditions in an indoor THz network. The nature of propagation in the proposed framework has been classified into LOS and NLOS propagation caused by human and wall blockages, for which probabilities for both types have been analytically derived. A multi-ray propagation model is employed where the channel model is characterized by large-scale fading (LSF) and small-scale fading (SSF) for both LOS and NLOS propagations. The path loss exponents (PLEs) have a major impact on coverage probability and have been taken into consideration in the analysis. Both the LOS and NLOS PLE have been considered. The mathematical expressions for mean interference, SINR coverage probability, and area spectral efficiency (ASE) have been derived.

The following sections of the research paper are organized as follows. Section II presents the system model for analysis. In Section III, an uplink interference analysis of the THz tier has been derived. In Section IV, a generalized expression for uplink coverage probability and ASE of a single-tier THz network have been derived. The analytical and simulation results have been presented in Section V. In the last Section VI, the conclusion of the research paper is presented.

## 2. System model

In Section II, we have presented the system model for a single-tier THz network and, using this model, derived the analytical expressions for network deployment, antenna models, blockages caused by humans and walls, the propagation model of the THz tier, along with the distance distribution association scheme.

### 2.1. THz network deployment

**Fig 1:**
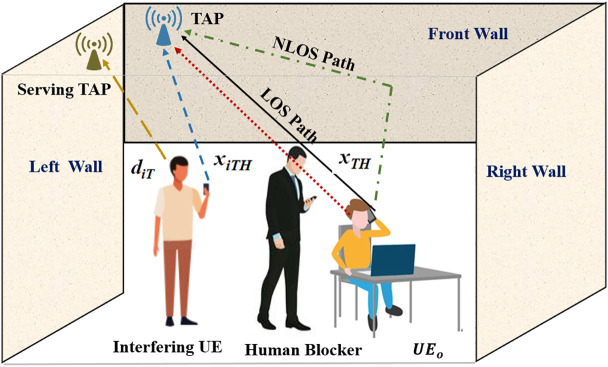
System model of a single-tier THz network depicting the presence of blockages and interferer UE.

In this paper, the TAPs location are modeled as an independent homogeneous PPP, represented by $\Phi_{TAP}$ with average node density denoted by $\lambda_{TAP}$, and are placed on the ceiling of the room with a fixed height $H_{TAP}$. The tagged user equipment ($UE_{o}$) is located at the origin in an indoor area. The location of $UE_{o}$ follows PPP, denoted by $\Phi_{UE}$, with average node density denoted by $\lambda_{UE}$. The $UE_{o}$ is assumed of fixed height $H_{UE}$, and it is also present in an indoor environment. The LOS path between the TAP and $UE_{o}$ is blocked by the blockages caused by humans and the wall as presented in Fig 1. The simulation parameters with symbols are summarized in Table 1.

**Table 1:** Symbols and simulation parameters.

Symbol	Simulation parameters
$\lambda_{TAP}$, $\lambda_{UE}$, $\lambda_{B}$, $\lambda_{W}$	TAP, UE, human and wall Blockage densities
$P_{UE}$	Transmitting power of UE
$H_{TAP}$, $H_{UE}$, $H_{B}$	Height of TAP, user, and human blocker
$f_{TAP}$	THz operating frequency
$\;K\left(f_{TAP}\right)$	THz molecular absorption coefficient
$\alpha_{TLOS}$, $\alpha_{TNLOS}$	LOS and NLOS PLEs for THz
$G_{TAP}$, $G_{UE}$	Gain of the main lobe of TAP and UE of 2D antenna model
$\theta_{m}$, $\theta_{s}$	Main lobe and side lobe with a beam widths of 2D antenna model
$G_{m,TAP}$, $G_{m,UE}$,	Main lobe antenna gain of TAP and UE of 3D antenna model
$G_{s,TAP}$, $\;G_{s,UE}$	Side lobe antenna gain of TAP and UE of 3D antenna model
$\;\varphi_{TAP,\text{\textbackslash{} }H}$, $\varphi_{TAP,\text{\textbackslash{} }V}$	Azimuth and vertical beam width of TAPs of 3D antenna model
$\varphi_{UE,\text{\textbackslash{} }H}$, $\varphi_{UE,\text{\textbackslash{} }V}$	Azimuth and vertical beam width of UEs of 3D antenna model

### 2.2. Antenna model

In this paper, we have considered two different dimensions of antenna models to analyze the impact of the THz uplink network performance in the presence of wall and human blockages. For a 2D antenna model, MC has been employed, which comprises of a single main lobe and several side lobes are directed at each other only in the horizontal direction. For simplicity, the same antenna gains have been considered for both TAPs and UEs.

Whereas, in a 3D antenna model, PS has been used to analyze the 3D beams at both UEs and TAPs. Since the transmission distance in THz communication is limited to only a few meters, it is necessary to employ both the horizontal and vertical heights. In this research, different antenna gains corresponding to both azimuth and vertical beam widths of UEs and TAPs are considered.

#### 2.2.1. Multi-cone antenna model.

The MC antenna model is employed to analyze the radiation patterns of the THz band. The antenna structure is depicted as MC antenna elements, consisting of a single main lobe and several side lobes $N_{k}$. Both the main lobe and side lobes are estimated as cone-shaped narrow beams [37] pointing in several directions. The same antenna has been considered for both TAPs and User Equipment (UEs). In the MC model, the gain for the single main lobe $G_{m}$ with beam width is $\theta_{m}$ and the antenna gain for all of the side lobes $G_{s}$ with equal beam width is $\theta_{s}$. The transmitter gain $G_{Tx}$ and receiving antenna gain $G_{Rx}$ are the same. The parameter $k=G_{s}/G_{m}$, where 0<k<1. The main lobe ${G_{m}}^{\text{2}}$ and side lobes $G_{s}$ of an antenna’s gains are expressed as,


$$\begin{array}{c} G_{m}=2{\left[\left(1-cos\frac{\theta_{m}}{\text{2}}\right)+k\text{\textbackslash{}hspace\{0.33em}\}N_{k}\left(1-cos\frac{\theta_{s}}{\text{2}}\right)\right]}^{-1} \end{array}$$ (1)

$$\begin{array}{c} G_{s}=k\text{\textbackslash{}hspace\{0.33em}\}G_{m} \end{array}$$ (2)

The main lobe of the tagged $UE_{o}$ to the tagged TAP at a distance $x_{TH}$ results in the square gain of the main lobes ${G_{m}}^{\text{2}}$ the transmitter antenna gain $G_{Tx}$ at the interfering UE, and the receiver antenna gain $G_{Rx}$ are arbitrarily selected from the discrete set $\left\{G_{m},G_{s},0\right\}$, denoted as,


$$\begin{array}{c} G_{i}=\left\{ \begin{array}{c} @l@G_{m},\text{\textbackslash{}hspace\{0.33em}\}with\text{\textbackslash{}hspace\{0.33em}\}probability,\text{\textbackslash{}hspace\{1em}\}P_{m}=\frac{\theta_{m}}{2\pi }\\ G_{s},with\text{\textbackslash{}hspace\{0.33em}\}probability,\text{\textbackslash{}hspace\{1em}\}P_{s}={\frac{N_{k}\text{\textbackslash{}hspace\{0.33em}}{\theta }}_{s}\}2\pi \\ 0,with\text{\textbackslash{}hspace\{0.33em}\}probability,\text{\textbackslash{}hspace\{1em}\}Pc=1-P_{m}-P_{s} \end{array}\right. \end{array}$$ (3)

The receiving and transmitting antenna gains are equal to the gain of the main lobe and side lobes with the probabilities $P_{TR}=P_{m}P_{s}$ respectively.

#### 2.2.2. Pyramidal-plus-sphere sectored antenna model.

The PS antenna model is employed to analyze the 3D radiation patterns of the THz band. The pyramid and sphere zones correspond to the main and side lobes of the antenna beam width [46–48]. According to the antenna theory principles, the antenna’s main lobe $G_{m,\rho }$ and the side lobes gains $G_{s,\rho }$ are represented as,


$$\begin{array}{c} G_{m,\rho }=\frac{P_{r,m}/\Psi_{m,\rho }}{P_{r,ms}/4\pi }=\frac{4\pi }{\left(k_{p}+1\right)\Psi_{m,\rho }} \end{array}$$ (4)

$$\begin{array}{c} G_{s,\rho }=\frac{P_{r,s}/\Psi_{s,\rho }}{P_{r,ms}/4\pi }=\frac{4\pi k_{p}}{\left(k_{p}+1\right)\left(4\pi -\Psi_{m,\rho }\right)} \end{array}$$ (5)

where $\rho \in \left\{T_{x},R_{x}\right\}$, $P_{r,m}$ and $P_{r,s}$ are the power of the main and side lodes, respectively, and $k_{p}=P_{r,s}/P_{r,m}$.


$$\begin{array}{c} P_{r,ms}=P_{r,m}+\;P_{r,s} \end{array}$$ (6)

The radiation of the antenna beam is modeled as a pyramidal shape with the azimuth beam width $\;\varphi_{\rho ,H}$ and the vertical beam width $\;\varphi_{\rho ,V}$. The angles corresponding to the main and side lodes are denoted by $\Psi_{m,\rho }$ and $\Psi_{s,\rho }$ is expressed as,


$$\begin{array}{c} \Psi_{m,\rho }=4arcsin\left(tan\left(\frac{\varphi_{\rho ,H}}{\text{2}}\right)tan\left(\frac{\varphi_{\rho ,V}}{\text{2}}\right)\right) \end{array}$$ (7)

$$\begin{array}{c} \Psi_{s,\rho }=4\pi -\Psi_{m,\rho } \end{array}$$ (8)

The signal originating from the main lobe of an interferer UE reaches TAP only when TAP lies in both the azimuth and vertical beam widths of the interferer UE. The probability of TAP lies with the azimuth beam width of the interfering UE $\;P_{\varphi_{UE,H}}$ is denoted as,


$$\begin{array}{c} P_{\varphi_{UE,H}}=\frac{\varphi_{UE,H}}{2\pi } \end{array}$$ (9)

The probability of TAP lies with the vertical beam width of the interferer UE $P_{\varphi_{UE,V}}$ is denoted as,


$$\begin{array}{c} P_{\varphi_{UE,V}=}\left\{ \begin{array}{c} @l@\gamma \left[cot^{\text{2}}\left(\xi -\frac{\varphi_{UE,V}}{2\pi }\right)-cot^{\text{2}}\left(\xi +\frac{\varphi_{UE,V}}{2\pi }\right)\right],\;0\leq x_{iTH}\leq x_{uv},\\ 1-\gamma \left[cot^{\text{2}}\left(\xi +\frac{\varphi_{UE,V}}{2\pi }\right)\right],\;\;x_{uv}\leq x_{iTH}\leq x_{vv}\\ 0,\;\;\;x_{iTH}\geq x_{vv} \end{array}\right. \end{array}$$ (10)

$$\begin{array}{c} \gamma =\frac{{\left(H_{TAP}-H_{UE}\right)}^{\text{2}}}{{R_{TH}}^{\text{2}}},\text{\textbackslash{}hspace\{0.33em}\}\xi =arctan\left(\frac{H_{TAP}-H_{UE}}{x_{iTH}}\right) \end{array}$$

$$\begin{array}{c} x_{uv}=\left(H_{TAP}-H_{UE}\right)cot\left(max\left[0,arctan\left(\frac{H_{TAP}-H_{UE}}{R_{TH}}-\frac{\varphi_{UE,V}}{\text{2}}\right)\right]\right), \end{array}$$

$$\begin{array}{c} x_{vv}=\left(H_{TAP}-H_{UE}\right)cot\left(min\left[\frac{\pi }{\text{2}},arctan\left(\frac{H_{TAP}-H_{UE}}{R_{TH}}-\frac{\varphi_{UE,V}}{\text{2}}\right)\right]\right) \end{array}$$

The signal originating from the main lobe of an interferer TAP reaches the UE only when the UE lies in both the azimuth and vertical beam widths of the interferer TAP. The probability of UE lies with the azimuth beam width of the interferer TAP $P_{\varphi_{TAP,H}}$ is denoted as,


$$\begin{array}{c} P_{\varphi_{TAP,H}}=\frac{\varphi_{TAP,H}}{2\pi } \end{array}$$ (11)

The probability of UE lies with the vertical beam width of the interferer TAP $P_{\varphi_{TAP,V}}$ is denoted as,


$$\begin{array}{c} P_{\varphi_{TAP,V}=}\left\{ \begin{array}{c} @l@\gamma \left[cot^{\text{2}}\left(\xi -\frac{\varphi_{TAP,V}}{2\pi }\right)-cot^{\text{2}}\left(\xi +\frac{\varphi_{TAP,V}}{2\pi }\right)\right],\;0\leq x_{iTH}\leq x_{ua},\\ 1-\gamma cot^{\text{2}}\left(\xi +\frac{\varphi_{TAP,V}}{2\pi }\right),\;x_{ua}\leq x_{iTH}\leq x_{va}\\ 0,\;x_{iTH}\geq x_{va} \end{array}\right. \end{array}$$ (12)

$$\begin{array}{c} x_{ua}=\left(H_{TAP}-H_{UE}\right)cot\left(max\left[0,arctan\left(\frac{H_{TAP}-H_{UE}}{R_{TH}}-\frac{\varphi_{TAP,V}}{\text{2}}\right)\right]\right), \end{array}$$

$$\begin{array}{c} x_{va}=\left(H_{TAP}-H_{UE}\right)cot\left(min\left[\frac{\pi }{\text{2}},arctan\left(\frac{H_{TAP}-H_{UE}}{R_{TH}}-\frac{\varphi_{TAP,V}}{\text{2}}\right)\right]\right) \end{array}$$

The directivity gains of interfering UE and TAP $G_{i}$ is expressed as,


$$\begin{array}{c} G_{i}=\left\{ \begin{array}{c} @l@G_{m,TAP}G_{m,UE\;}\text{with\textbackslash{} probability\textbackslash{} \textbackslash{} }\alpha_{TAP}\beta_{UE}\\ G_{m,TAP}G_{s,UE}\text{\textbackslash{} with\textbackslash{} probability}\;\alpha_{TAP}\left(1-\beta_{UE}\right)\\ G_{s,TAP}G_{m,UE\;}\text{with\textbackslash{} probability\textbackslash{} }\left(1-\alpha_{TAP}\right)\beta_{UE}\text{\textbackslash{} }\\ G_{s,TAP}G_{s,UE}\text{\textbackslash{} with\textbackslash{} probability\textbackslash{} }\left(1-\alpha_{TAP}\right)\left(1-\beta_{UE}\right) \end{array}\right. \end{array}$$ (13)

$$\begin{array}{c} \alpha_{TAP}=P_{\varphi_{TAP,V}}P_{\varphi_{TAP,H}},\text{\textbackslash{}hspace\{0.33em}\}\beta_{UE}=P_{\varphi_{UE,V}}P_{\varphi_{UE,H}} \end{array}$$

where $G_{m,TAP}$ and is the main lobe antenna gain of TAP, $G_{m,UE}$ is the main lobe antenna gain of UE, $G_{s,TAP}$ is the side lobes antenna gain of TAP, and $G_{s,UE}$ is the side lobes antenna gain of UE. The receiving and transmitting antenna gains of the main lobe and side lobes, with the probabilities, $P_{TR}$ are expressed as $G_{i}\in G_{m,TAP}G_{m,UE},G_{m,TAP}G_{s,UE},G_{s,TAP}G_{m,UE},G_{s,TAP}G_{s,UE}$.

### 2.3. Human blockages

**Fig 2:**
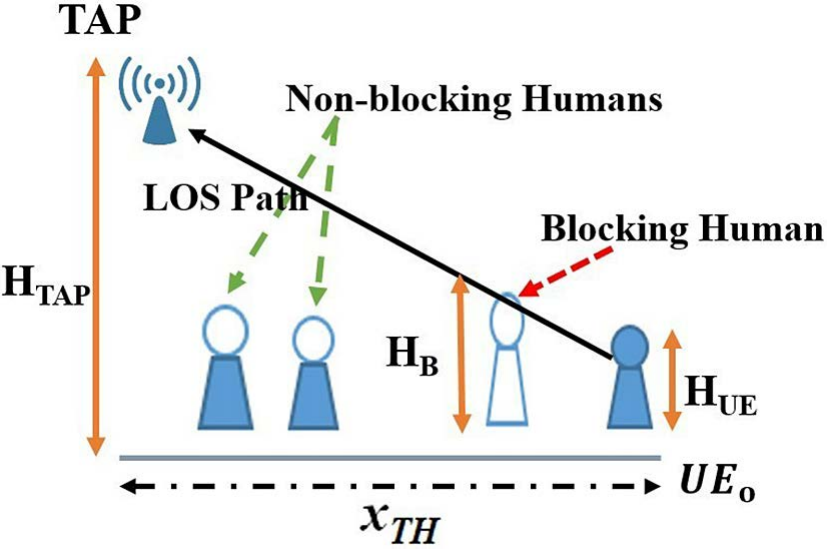
Side view of a LOS path in the presence of a human blockage.

THz communications are highly susceptible to human blockages. The blockage that occurs between $UE_{o}$ and the TAP link is caused by dynamic human blockages [49]. The human blockers are considered as a cylinder with a height of $H_{B}$ and radius of $R_{B}$, and their location complies with another PPP with the density of $\lambda_{B}$ as demonstrated in Fig 2.

**Fig 3:**
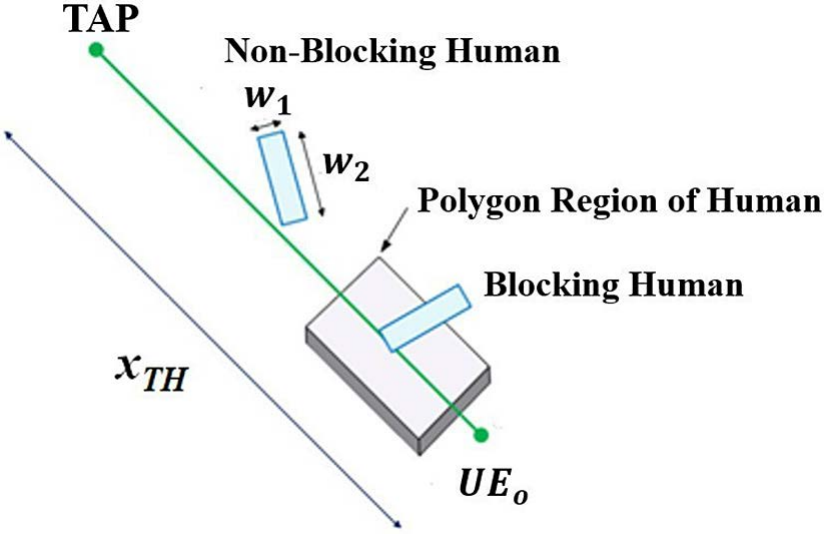
Top view of a LOS path in the presence of a human blocker.


Similarly, the authors of [26] suggested that the area in which dynamic human blockers appear can be approximated by a polygon. Let us consider a specific instant of time, in which a human blockage blocks the LOS link between TAP and $UE_{o}$ within the specified area known as the polygon region, as shown in Fig 3. The human blockages have been modelled by considering rectangular absorbing screens [50,51] it is also called as DKE model. Where polygon region widths are denoted by $w_{\text{1}}$ and $w_{\text{2}}$ respectively. Moreover, the mobility of the human blockages [52] supports the random directional model. It is assumed that $\;H_{TAP}>H_{B}>H_{UE}$. The human blockages are homogenously distributed between 0 and 2π. The $UE_{o}$ is assumed to be stationary. The number of human blockages that block the LOS path between TAP and $UE_{o}$ is given as,

$$\begin{array}{c} \beta H=\left(w_{\text{1}}w_{\text{2}}+\frac{\text{2}}{\pi }\left(w_{\text{1}}+w_{\text{2}}\right)\frac{\left(H_{B}-H_{UE}\right)}{\left(H_{TAP}-H_{UE}\right)}x_{TH}\right)\lambda_{B} \end{array}$$ (14)

Therefore, the LOS and NLOS probability function is respectively expressed as,


$$\begin{array}{c} P_{HB,LOS}\left(x_{TH}\right)=e^{-\beta H\;x_{TH}} \end{array}$$ (15)

$$\begin{array}{c} P_{HB,NLOS}\left(x_{TH}\right)=1-e^{-\beta H\;x_{TH}} \end{array}$$ (16)

### 2.4. Wall blockages

**Fig 4:**
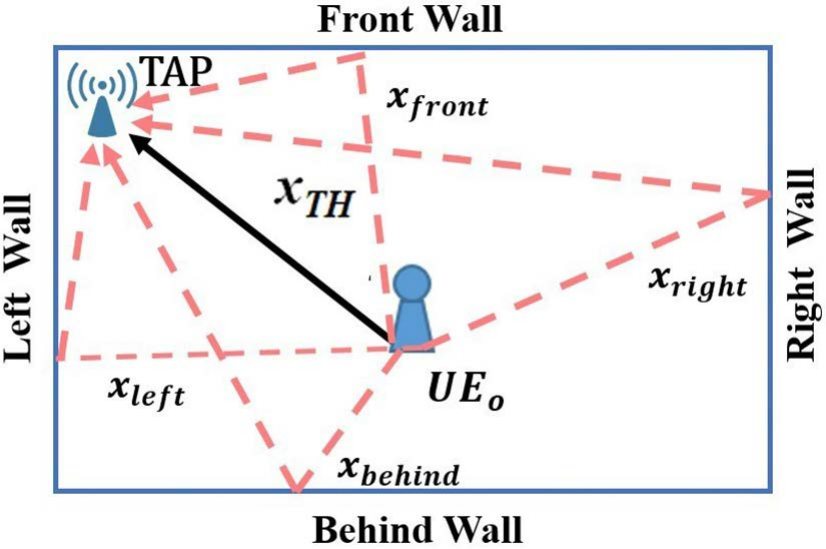
Rectangle geometry of an indoor room.


THz bands are also highly susceptible to wall blockages. In this regard, the MPLPs have been considered [27] to model the wall blockages. The room walls are formed as two independent MPLPs, where midpoints are distributed for each 1D PPP expressed as $\Phi_{x}$ and $\Phi_{y}$, with the density denoted as $\lambda_{W}$. The TAP associated with $UE_{o}$ and interfering UEs are located in the same room. The transmission between $UE_{o}$ and TAP is blocked if a wall obstructs them. The probability of the wall blockages [37], for an arbitrary link between $UE_{o}$ - TAP, is expressed as


$$\begin{array}{c} P_{WB}\left(x_{TH},\theta \right)=1-\text{\textbackslash{}hspace\{0.33em}\}e^{-\lambda_{w}x_{TH}\left(\left|cos\theta \right|\text{\textbackslash{}hspace\{0.33em}\}+\text{\textbackslash{}hspace\{0.33em}\}\left|sin\theta \right|\right)} \end{array}$$ (17)

where $x_{TH}$ denotes the 2D distance between the link $UE_{o}$ and TAP, θ denotes the azimuth angle, and $P_{WB}$ complies with the void probability of PPPs. To provide the tractability of coverage analysis, the wall-blocking probability $P_{WB}\left(x_{TH},\theta \right)$ is proposed by [53], which is denoted as $STR\left(x_{TH}\right)$, which is equivalent to 0 when $0<x_{TH}<R_{TH}$ and 1 otherwise. The rectangular interference region is approximated as a circular region while considering the wall-blocking probability [28], as presented in Fig 4. The average number of UEs in the rectangular region is expressed as,

$$\begin{array}{c} \sigma_{R}=𝔼\;\left[\lambda_{UE}\;\left(x\;right+x\;left\right)\text{\textbackslash{}hspace\{0.33em}\}\left(ybehind+yfront\right)\right]\text{\textbackslash{}hspace\{0.33em}\;\}={\frac{4\text{\textbackslash{}hspace\{0.33em}}{\lambda }}_{UE}\}{\lambda_{W}}^{\text{2}} \end{array}$$ (18)

where the 2D distances between a user and the walls are expressed as xright, xleft, ybehind, and yfront, respectively.

**Fig 5:**
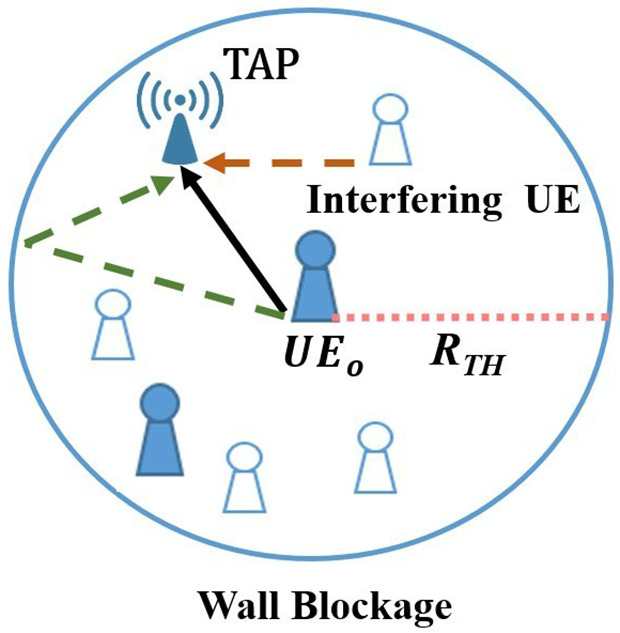
Equivalent circle region of an indoor environment.


In the THz network, a constant circle region with a fixed radius $R_{TH}$ can be calculated with the propagation model presented in [31,54]. Therefore, the average number of UEs in the circular region, as presented in Fig 5, is derived as,

$$\begin{array}{c} \sigma_{C}=\pi {R_{TH}}^{\text{2}}\lambda_{UE} \end{array}$$ (19)

whereas Equation (19) results from the mean of the Poisson distribution. Therefore, due to the criteria of mean APs $\sigma_{R}=\sigma_{C}$. The radius of the circular region of an indoor room is denoted as,


$$\begin{array}{c} R_{TH}=\frac{\text{2}}{\lambda_{W}\sqrt{\pi }} \end{array}$$ (20)

### 2.5. Blockage effects on the THz band

The LOS link is blocked due to the high penetration losses in the THz band. The $UE_{o}$ can only communicate with the TAP via NLOS links. Therefore, when the blockage is caused by the human body, the transmission of the signal can be done with NLOS links that are reflected on the room walls where the TAP and the $UE_{o}$ receiver reside. Similarly, if the blockage is caused by the wall, where TAP and $UE_{o}$ are present in different rooms, then the transmission of the signal is considered negligible. Hence, there is no transmission through NLOS links in wall blockages. The LOS probability is given by,


$$\begin{array}{c} P_{LOS}\left(x_{TH}\right)=\left(1-STR\left(x_{TH}\right)\right)\left(P_{HB,LOS}\left(x_{TH}\right)\right)=e^{-\beta Hx_{TH}} \end{array}$$ (21)

The NLOS probability is denoted as,


$$\begin{array}{c} P_{NLOS}\left(x_{TH}\right)=\left(1-STR\left(x_{TH}\right)\right)\left(P_{HB,NLOS}\left(x_{TH}\right)\right)=1-e^{-\beta H\;x_{TH}} \end{array}$$ (22)

For $x_{TH}<R_{TH}$, where $x_{TH}$ is the distance between the TAP and $\;UE_{o}$.

### 2.6. Propagation model of THz tier

The power loss $PW_{loss}$ of the THz wave is determined by the LSF $Lf_{THz}$ and SSF $Sf_{THz}$. In a THz tier [55], the received signal power at the TAPs from $UE_{o}$ at a distance $x_{TH}$ can be stated as,


$$\begin{array}{c} PW_{loss}\left(x_{TH}\right)=Sf_{THz}\left(x_{TH}\right)Lf_{THz}\left(x_{TH}\right) \end{array}$$ (23)

Therefore, the LSF for the LOS $Lf_{THz,LOS}$ is expressed as,


$$\begin{array}{c} Lf_{THz,LOS}\text{\textbackslash{} }={\left(\frac{c}{4\pi f_{TAP}}\right)}^{\text{2}}{x_{TH}}^{-\alpha_{TLOS}}e^{-K\left(f_{TAP}\right)\;x_{TH}}\;{x_{TH}}^{\alpha_{TLOS}\;\tau } \end{array}$$ (24)

The LSF for the NLOS $Lf_{THz,NLOS}$ is expressed as,


$$\begin{array}{c} \;Lf_{THz,NLOS}={\left(\frac{c}{4\pi f_{TAP}}\right)}^{\text{2}}{x_{TH}}^{-\alpha_{TNLOS}}e^{-K\left(f_{TAP}\right)x_{TH}}{x_{TH}}^{\alpha_{TNLOS}\;\tau }\text{\textbackslash{}hspace\{0.33em}\}𝔼\left[{R_{C}}^{\text{2}}\right] \end{array}$$ (25)

where $K\left(f_{TAP}\right)$ the absorption coefficient depends on the operating frequency $f_{TAP}$, τ is the power control factor. $R_{C}$ denoted as the reflection coefficient based on the wall material and frequency. It is modeled as an independent normal random variable $\;R_{C}\left[dB\right]\sim \mathbb{N}\left(\mu_{RC},\text{\textbackslash{}hspace\{0.33em}\}{\sigma_{RC}}^{\text{2}}\right)$. The second moment of reflection coefficient is expressed as,


$$\begin{array}{c} \;𝔼\left[{R_{C}}^{\text{2}}\right]=exp\left(2\frac{ln10}{10}\text{\textbackslash{}hspace\{0.33em}\}\mu_{RC}+\text{\textbackslash{}hspace\{0.33em}\}2\text{\textbackslash{}hspace\{0.33em}\}{\left(\frac{ln10}{10}\text{\textbackslash{}hspace\{0.33em}\}\sigma_{RC}\right)}^{\text{2}}\right) \end{array}$$ (26)

The LOS and NLOS paths induced by blockage are distinguished by different PLEs $\alpha_{TLOS}$ and $\alpha_{TNLOS}$. For the LOS [28], the Gamma distribution and the NLOS exponential distribution are assumed. The multi-ray propagation model for a single-tier THz wave is stated as an integration of LOS and $N_{NLOS}$ waves reflected by the room walls, denoted as,


$$\begin{array}{c} \begin{array}{c} PW\left(x_{TH}\right)=Sf_{THz,LOS}\left(x_{TH}\right)\text{\textbackslash{}hspace\{0.33em}\}Lf_{THz,LOS}\left(x_{TH}\right)\text{\textbackslash{}hspace\{0.33em}\}I_{LOS}\\ +\overset{N_{NLOS}}{\underset{i=1}{\sum }}\text{\textbackslash{}hspace\{0.33em}\}Sf_{THz,NLOS}\left(x_{TH}\right)\text{\textbackslash{}hspace\{0.33em}\}Lf_{THz,NLOS}\left(x_{TH}\right) \end{array} \end{array}$$ (27)

where $I_{LOS}$ is an indicator notation that is equivalent to 1, when the LOS link between the TAP and $UE_{o}$ is blocked. In $Sf_{THz,LOS}$, Gamma random variable with a shape parameter $\alpha \left(x_{TH}\right)$ and the rate parameter $\beta \left(x_{TH}\right)$, whereas in $Sf_{THz,NLOS}$ the exponential distribution with the rate parameter $\mu \left(x_{TH}\right)$ are employed. The parameters are expressed as,


$$\begin{array}{c} \alpha \left(x_{TH}\right)=\frac{𝔼{\left[PW\left(x_{TH}\right)\right]}^{\text{2}}}{Var\left(PW\left(x_{TH}\right)\right)} \end{array}$$ (28)

$$\begin{array}{c} \beta \left(x_{TH}\right)=Lf_{THz,LOS}\left(x_{TH}\right)\text{\textbackslash{}hspace\{0.33em}\}\frac{𝔼\left[PW\left(x_{TH}\right)\right]}{Var\left(PW\left(x_{TH}\right)\right)} \end{array}$$ (29)

$$\begin{array}{c} \mu \left(x_{TH}\right)\frac{L\;f_{THz,NLOS}\left(x_{TH}\right)}{𝔼\left[PW\left(x_{TH}\right)\right]} \end{array}$$ (30)

### 2.7. Distance distribution of the nearest-TAP user association scheme

The $UE_{o}$ selects the nearest TAP to link with, i.e., the serving TAP is selected based on minimum distance, and the interfering TAP is all further than the associated TAP. The probability density function (PDF) of $x_{TH}$ is discussed by [56],


$$\begin{array}{c} f_{AP,UE}=2\pi \lambda_{TAP}x_{TH}e^{-\pi \;\lambda_{TAP}\;{x_{TH}}^{\text{2}}},\;0\leq x_{TH}\leq R_{TH} \end{array}$$ (31)

The conditional distribution of the distance from $UE_{o}$ to the nearest TAP denoted by $fd_{TAP}$ is expressed as,


$$\begin{array}{c} fd_{TAP}=\frac{2\pi \lambda_{TAP}}{1-e^{-\pi \lambda_{TAP}{R_{TH}}^{\text{2}}}}x_{TH}\text{\textbackslash{}hspace\{0.33em}\}e^{-\pi \lambda_{TAP}{x_{TH}}^{\text{2}}} \end{array}$$ (32)

## 3. Uplink interference analysis

In Section III, we have derived the uplink interference analysis of a single-tier THz network in the presence of human and wall blockages. The received uplink SINR at the tagged TAP at a distance $x_{TH}$ from a $UE_{o}$ can be expressed as,


$$\begin{array}{c} SINR_{THz}={\frac{P_{UE}\text{\textbackslash{}hspace\{0.33em}}{G}}_{TAP}G_{UE}\text{\textbackslash{}hspace\{0.33em}\}Sf_{THz}\left(x_{TH}\right)\text{\textbackslash{}hspace\{0.33em}\}Lf_{THz}\left(x_{TH}\right)\}I_{agg,THz}\text{\textbackslash{}hspace\{0.33em}+\text{\textbackslash{}hspace\{0.33em}\}{\sigma^{\text{2}}}_{Noise}\} \end{array}$$ (33)

where $P_{UE}$ is the transmit power of $UE_{o}$,$G_{TAP}$,$G_{UE}$ are the antenna gain of $UE_{o}$ and TAP. ${\sigma^{\text{2}}}_{Noise}$ is the noise power, and $\;I_{agg,THz}$ is the aggregate interference power of the THz tier and it is expressed as,


$$\begin{array}{c} {I_{agg,THz}=\sum }_{x_{iTH}\in UE\;int}P_{UE}G_{i}S\;f_{THz}\left(x_{iTH}\right)\;L\;f_{THz}\left(x_{iTH}\right) \end{array}$$ (34)

Proof has been provided in Appendix A.

The final expression for aggregate interference power is given as,


$$\begin{array}{c} \begin{array}{c} {ℒ}_{I_{agg,THz}}\left(s\right)=exp(-2\pi \lambda_{TAP}P_{TR}\overset{R_{TH}}{\underset{x_{TH}}{\int }}\left(1-{\left(1-\frac{s_{TL}P_{UE}G_{TAP}G_{UE}Lf_{THz,LOS}\left(x_{TH}\right)}{\beta \left(x_{TH}\right)}\right)}^{-\alpha \left(x_{TH}\right)}\right)P_{LOS}\left(x_{TH}\right)\\ +\left(1-\left(-\frac{\mu \left(x_{TH}\right)}{\mu \left(x\right)-s_{TNL}P_{UE}G_{TAP}G_{UE}Lf_{THz,NLOS}\left(x_{TH}\right)}\right)\right)P_{NLOS}\left(x_{TH}\right))\;fd_{TAP}\text{\textbackslash{}hspace\{0.33em}\}x_{TH}dx_{TH}\text{\textbackslash{}hspace\{0.33em}\}\; \end{array} \end{array}$$ (35)

The mean interference power (MIP) is expressed as,


$$\begin{array}{c} \begin{array}{c} 𝔼\left[I_{agg{,}_{THz}}\right]=\left(4\pi^{\text{3}}{\lambda_{TAP}}^{\text{3}}P_{TR}P_{UE}G_{TAP}G_{UE}\right)\;\overset{R_{TH}}{\underset{\text{0}}{\int }}\overset{R_{TH}}{\underset{x_{TH}}{\int }}(\frac{\alpha \left(x_{TH}\right)}{\beta \left(x_{TH}\right)}Lf_{THz,LOS}\left(x_{TH}\right)P_{LOS}\left(x_{TH}\right)\\ +\frac{Lf_{THz,NLOS}\left(x_{TH}\right)}{\mu \left(x_{TH}\right)}P_{NLOS}\left(x_{TH}\right))x_{TH}dx_{TH}xe^{-{\lambda_{TAP}}^{\text{2}}\pi^{\text{2}}x^{\text{3}}}dx \end{array} \end{array}$$ (36)

## 4. Uplink coverage probability and area spectral efficiency analysis

In Section IV, we have investigated the uplink coverage probability (UCP) and area spectral efficiency (ASE) to analyze the single-tier THz network in the presence of human and wall blockages. The total uplink coverage probability of the THz Tier$\;P_{C}\left(T\right)$ can be stated as,


$$\begin{array}{c} P_{C}\left(T\right)=\overset{R_{TH}}{\underset{\text{0}}{\int }}\left(P_{C,TLOS}\left(T\right)\text{\textbackslash{}hspace\{0.33em}\}P_{LOS}\left(x_{TH}\right)+P_{C,TNLOS}\left(T\right)P_{NLOS}\left(x_{TH}\right)\right)f_{AP,UE}\left(x_{TH}\right)dx_{TH} \end{array}$$ (37)

Proof has been provided in Appendix B.

The area spectral efficiency (ASE) is a measure of the total data rate per unit area, normalized by the bandwidth and it evaluates the total network throughput. ASE is measured in [bps/Hz/m^2^] and it is expressed as,


$$\begin{array}{c} \;ASE=\lambda_{TAP}{log}_{\text{2}}\left(1+T\right)P_{C}\left(T\right) \end{array}$$ (38)

## 5. Results and discussion

In Section V, the analytical and simulation results for aggregated interference, MIP derived in Section III, UCP, and ASE derived in Section IV have been elaborated. The analytical results obtained from Equations (35), (36), (37), and (38) have been validated by Monte Carlo simulations and are found in agreement. For attaining analytical and simulation results, the operating frequency of THz is set as $f_{TAP}$ = 1 THz and the molecular absorption coefficient of THz $\;K\left(f_{TAP}\right)$ = 0.05/m. The transmitting power of $\;UE_{o}$ is set as $P_{UE}$ = 23dBm, and the power control factor is set as τ = 0 have been assumed. Here, Additive white Gaussian noise is set as ${\sigma^{\text{2}}}_{Noise}$ = -84dBm. The reflection coefficient parameters are set as $\mu_{RC}$ = -5dB, and $\sigma_{RC}$ = 2 dB. The TAP, human blocker, and wall blockage densities are set as $\lambda_{TAP}$ = 0.1/m^2^, $\lambda_{B}$ = 0.2/m^2^, and $\lambda_{W}$ = 0.1/m. Whereas to investigate the impact of 3D antenna and blockage effects, the height of TAP, $\;UE_{o}$, and human blockers are set as $H_{TAP}$ = 3m, $H_{UE}$ ={0.9m, 1.3m}, and $H_{B}$ = 1.7m [44,45]. The polygon region widths are considered as $w_{\text{1}}$ = 0.6m and $w_{\text{2}}$ = 2.6m. The LOS and NLOS PLEs for THz, $\alpha_{TLOS}$ = {2, 3} and $\alpha_{TNLOS}$ ={4, 5}. Moreover, to investigate the impact of the 2D MC antenna model, the gain of the main lobe of TAP and UE are set as $G_{TAP}$ =$G_{UE}$ = {11.6dB, 17dB, 21.6dB} respectively. The main lobe with a beam width and the side lobe with a beam width, the number of side lobes, and the ratio of the side lobe with the main lobe are set as, $\theta_{m}$ are 60°, 30°, 15°, $\theta_{s}$ is 15°, $N_{k}$ = 12, k = 0.05, respectively. Whereas, to investigate the impact of 3D PS antenna model $G_{m,TAP}$ = {25dB, 25dB, 22.5dB}, $G_{m,UE}$ = {15dB, 12.5dB, 15dB}, $G_{s,TAP}$ = $\;G_{s,UE}$ = -10dB. The azimuth beam width $\;\varphi_{TAP,\text{\textbackslash{} }H}$ and vertical beam width $\varphi_{TAP,\text{\textbackslash{} }V}$ of TAPs are {10°, 10°, 15°}. Furthermore, the azimuth beam width $\varphi_{UE,\text{\textbackslash{} }H}$ and vertical beam width $\varphi_{UE,\text{\textbackslash{} }V}$ of UEs are {33°, 48°, 33°}.

**Fig 6:**
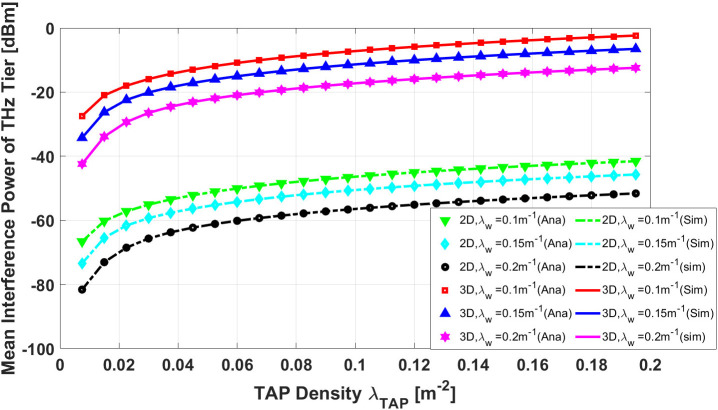
MIP of the THz tier vs. TAP density $\lambda_{TAP}$ for different values of wall blockage densities $\lambda_{W}$.

The MIP of the THz tier versus the TAP density $\lambda_{TAP}$ for different values of wall blockage densities $\lambda_{W}$ are shown in Fig 6. Here, both the 2D antenna model ($G_{TAP}$ =$G_{UE}$ = 21.6dB) and 3D antenna model ($G_{m,TAP}$ 25dB, $G_{m,UE}$ = 15dB) have been employed. The PLEs are set as $\alpha_{TLOS}$ = 2 and $\alpha_{TNLOS}$ = 4. The results are attained by considering three different values of the wall blockage densities $\lambda_{W}$ are 0.1m^−1^, 0.15m^−1^, 0.2m^−1^ respectively. The result indicate that TAP density becomes greater, the interference power increases. Hence, MIP is directly proportional to the$\lambda_{TAP}$. Moreover, it is also observed that as the wall blockage density increases, the MIP decreases with the increase of TAP density due to reduced LOS paths. Furthermore, it is also observed that the MIP of the 3D antenna model is higher than 2D antenna model, because in the 3D antenna model, the interferer exists in both vertical and horizontal directions, thereby increasing the interfering links.

**Fig 7:**
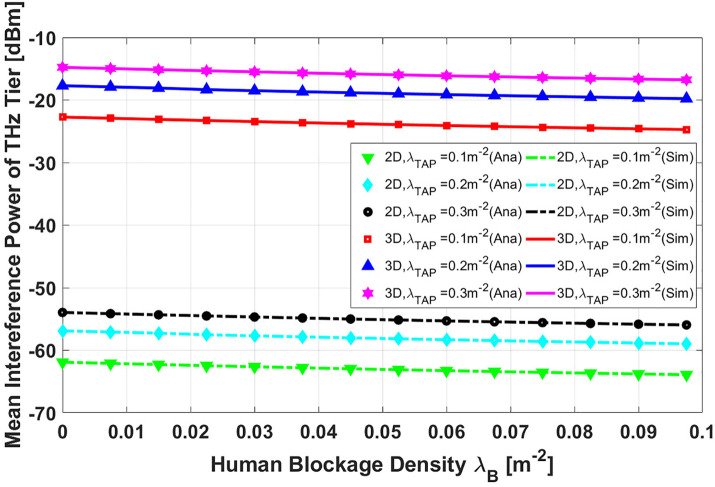
MIP of the THz tier vs. human blockage density $\lambda_{B}$ for different values of TAP densities $\lambda_{TAP}$.

The MIP of the THz tier against the human blockage density $\lambda_{B}$ for different values of TAP densities $\lambda_{TAP}$ are shown in Fig 7. Here 2D antenna model ($G_{TAP}$ =$G_{UE}$ = 21.6dB) and 3D antenna model ($G_{m,TAP}$ = 25dB, $G_{m,UE}$ = 15dB) have been employed. The PLEs are set as set as $\alpha_{TLOS}$ = 2 and $\alpha_{TNLOS}$ = 4. The results are attained by considering three different values of the TAP densities $\lambda_{TAP}$ are 0.1m^−2^, 0.2m^−2^, 0.3m^−2^ respectively. The result indicates that as the human blockage density becomes larger, the MIP decreases. It is due to the interfering signal probability being blocked by the human blockage becomes high. Moreover, it is also observed that as the TAP density increases, the MIP also increases with the increase in human blockage density. This is due to reducing LOS probability, and higher TAP density raises the MIP. Furthermore, it is noticed that the MIP of the 3D antenna model is higher than 2D antenna model, because the 3D antenna model captures vertical directions also, which increases the interfering links between the TAP and UE.

**Fig 8:**
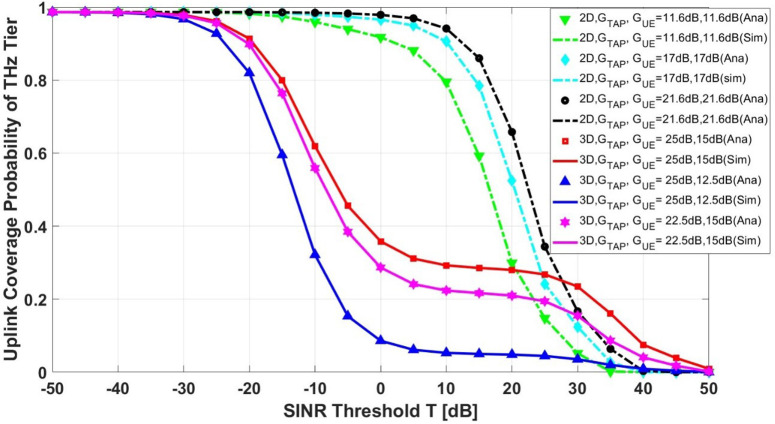
UCP of THz tier vs. SINR threshold T (dB) for different 2D and 3D antenna gains.

Fig 8 illustrates the UCP of the THz tier against the SINR threshold T (dB) for various antenna gains of 2D and 3D antenna models of both TAP and UE. Here, the gains of the 2D antenna model are set as $G_{TAP}$ =$G_{UE}$ = {11.6dB, 17dB, 21.6dB}, and the gains of the 3D antenna model are set as $G_{m,TAP}$ = {25dB, 25dB, 22.5dB}, $G_{m,UE}$ ={15dB, 12.5dB, 15dB}, respectively. The PLEs are set as $\alpha_{TLOS}$ = 2 and $\alpha_{TNLOS}$ = 4. It is observed that as the SINR Threshold T (dB) increases, the UCP decreases. As the T (dB) increases, interference becomes the main limiting factor, and the tagged TAP cannot receive coverage at a higher SINR. Moreover, the narrow beam width and higher gains of the 2D and 3D antenna models improve the UCP because its impact on the received signal is higher than the interference. However, as evident from Fig 8, the UCP of the 3D antenna model is lower than 2D antenna model. Because the 2D antenna model is oversimplified, it considers only the horizontal direction, and it underestimates the interference compared to the 3D antenna model. At a 30dB SINR threshold level, both 2D and 3D antenna models result in showing nearly similar UCP. This is due to the LOS path between UE-TAP, even though both models handle interference differently.

**Fig 9:**
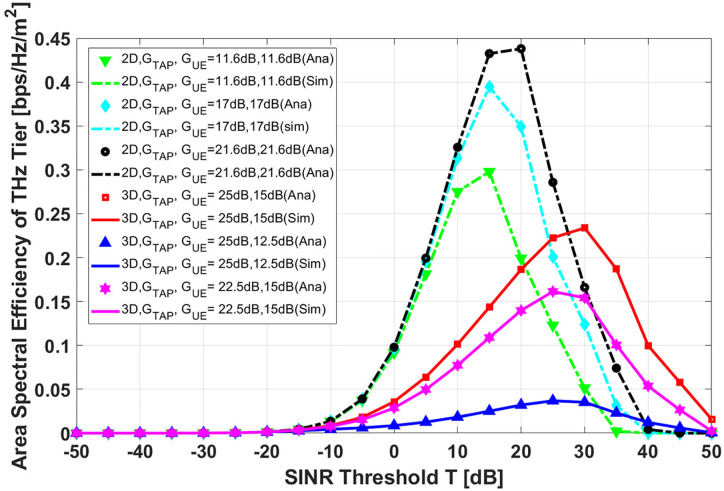
ASE of THz tier vs. T (dB) for different 2D and 3D antenna gains.

The ASE of the THz tier versus the T (dB) is illustrated in Fig 9, for different antenna gains of 2D and 3D models of both TAP and UE. Here, the antenna gain of the 2D model are set as $G_{TAP}$ = $G_{UE}$ = {11.6dB, 17dB, 21.6dB}, and the gain of the 3D antenna model are set as $G_{m,TAP}$ = {25dB, 25dB, 22.5dB}, $G_{m,UE}$ ={15dB, 12.5dB, 15dB}, respectively. The PLEs are set as $\alpha_{TLOS}$ = 2 and $\alpha_{TNLOS}$ = 4. It is observed that as the lower T (dB), the ASE occurs. Furthermore, the narrow beam width and higher gains of the 2D and 3D antenna models show a better growth rate. Moreover, it is observed that the ASE of the 3D antenna model experiences a lower growth rate than the 2D antenna model. A 2D antenna model accounts for horizontal directions and ignores the impact of the vertical directions. Therefore, a 3D antenna model shows a more realistic representation of ASE with different antenna gains of both TAP and UE.

**Fig 10:**
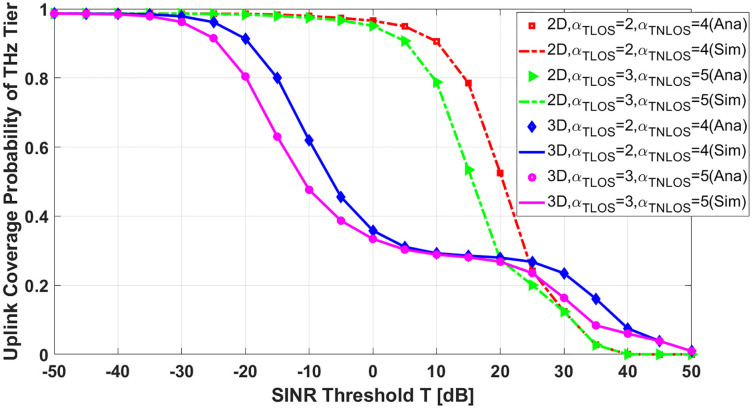
UCP of THz tier vs. T (dB) for PLEs $\alpha_{TLOS}$ ={2, 3} and $\alpha_{TNLOS}$ = {4, 5}.

Fig 10, shows the UCP of the THz tier vs the T (dB) for various values of the PLEs $\alpha_{TLOS}$ ={2,4} and $\alpha_{TNLOS}$ ={3,5}, respectively. Here, both the antenna gain of the 2D model ($G_{TAP}$ =$G_{UE}$ = 21.6dB) and antenna gain of the 3D model ($G_{m,TAP}$ = 25dB, $G_{m,UE}$ = 15dB) have been employed. It is noted that as the SINR threshold T (dB) increases, the UCP decreases. Also, the UCP improves at the higher SINR for PLEs $\alpha_{TLOS}$ = 2 and $\alpha_{TNLOS}$ = 4. Moreover, as evident from Fig 10, the UCP of the 3D antenna model is lower than 2D antenna model. At a 20dB SINR threshold level, the UCP of the 3D antenna model results in nearly the same UCP as the 2D antenna model with PLEs $\alpha_{TLOS}$ = 3 and $\alpha_{TNLOS}$ = 5. This is due to the limited vertical interference of the 3D antenna model at a moderate SINR threshold.

**Fig 11:**
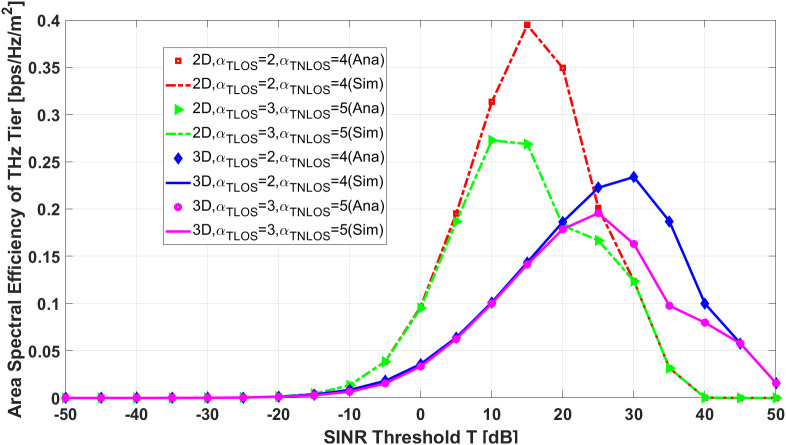
ASE of THz tier vs. T (dB) for PLEs $\alpha_{TLOS}$ ={2, 3} and $\alpha_{TNLOS}$ = {4, 5}.

The ASE of the THz tier against the T (dB) is illustrated in Fig 11, for various values of the PLEs $\alpha_{TLOS}$ ={2, 3} and $\alpha_{TNLOS}$ = {4, 5} respectively both the antenna gain of the 2D model ($G_{TAP}$ =$G_{UE}$ = 21.6dB) and antenna gain of the 3D model ($G_{m,TAP}$ = 25dB, $G_{m,UE}$ = 15dB) have been employed. It is observed that as the lower T (dB), the ASE occurs. Also, the ASE for PLEs $\alpha_{TLOS}$ = 2 and $\alpha_{TNLOS}$ = 4 shows a better growth rate than PLEs $\alpha_{TLOS}$ = 3 and $\alpha_{TNLOS}$ = 5. Moreover, it is observed that the ASE of the 3D antenna model experiences a lower growth rate than the 2D antenna model. At a 20dB SINR threshold level, the ASE of the 3D antenna model results in nearly the same ASE as the 2D antenna model with PLEs $\alpha_{TLOS}$ = 3, and $\alpha_{TNLOS}$ = 5. It is due to the minimal effects of vertical interference in the 3D antenna model under different PLE conditions.

**Fig 12:**
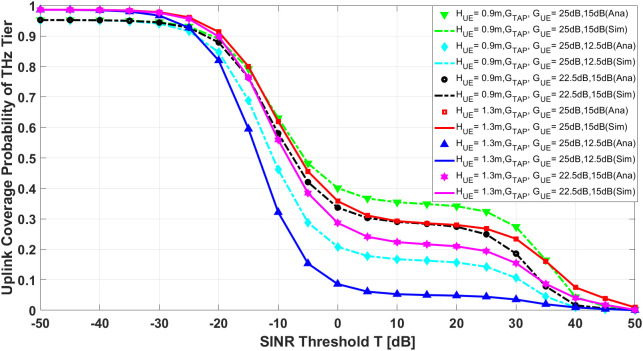
UCP of THz tier vs. T (dB) for$H_{UE}$ ={0.9m, 1.3m} considering various 3D antenna gains.

Fig 12 illustrates the UCP of the THz tier against the SINR threshold T (dB) for different $H_{UE}$ = {0.9m, 1.3m}, for various antenna gains of 3D antenna models of both TAP and UE. Here, the gains of the 3D antenna model are set as $G_{m,TAP}$ = {25dB, 25dB, 22.5dB}, $G_{m,UE}$ = {15dB, 12.5dB, 15dB}, respectively. The PLEs are set as $\alpha_{TLOS}$ = 2 and $\alpha_{TNLOS}$ = 4. It is noted that as the SINR Threshold T (dB) increases, the UCP decreases. As the T (dB) increases, interference becomes the main limiting factor, and the tagged TAP cannot receive coverage at a higher SINR. However, as evident from Fig 12, the UCP by employing $H_{UE}$ = 1.3m, UE performs better at low SINR due to the improved LOS probability and better elevation alignment of the antenna. Conversely, at $H_{UE}$ = 0.9m, the UCP performs better at higher SINR, because it is more likely to be blocked by human blockage, which reduces interference from other users, thereby improves the coverage. Furthermore, in the mid-SINR range (T (dB) = −10–30 dB), the UCP at $H_{UE}$ = 0.9m shows better performance. It is because of higher gain produces narrower beam widths, making the LOS more sensitive to blockages while effectively suppressing interference.

**Fig 13:**
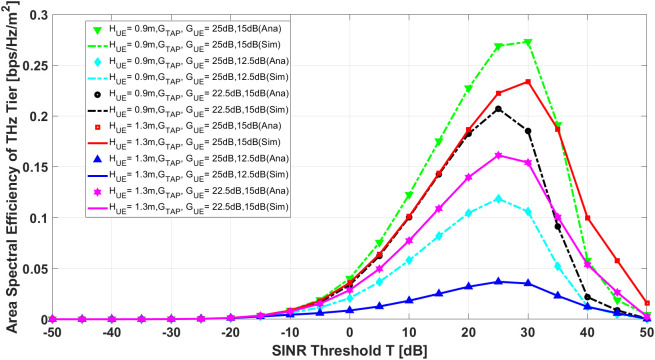
ASE of THz tier vs. T (dB) for$H_{UE}$ ={0.9m, 1.3m} considering various 3D antenna gains.

The ASE of the THz tier versus the T (dB) is illustrated in Fig 13, for different $H_{UE}$ = {0.9m, 1.3m}, for various antenna gains of 3D antenna models of both TAP and UE. Here, the gains of the 3D antenna model are set as $G_{m,TAP}$ = {25dB, 25dB, 22.5dB}, $G_{m,UE}$ = {15dB, 12.5dB, 15dB}, respectively. The PLEs are set as $\alpha_{TLOS}$ = 2 and $\alpha_{TNLOS}$ = 4. It is observed that as the lower T (dB), the ASE occurs. Furthermore, the ASE at $H_{UE}$ = 0.9m provides better a better growth rate than $H_{UE}$ = 1.3m, because the UE at lower height may experiences reduced interference from the other UEs. Additionally, in the mid-SINR range (T (dB) = 10–30 dB), $H_{UE}$ = 1.3m may increase interference or reduce beam alignment efficiency, whereas $H_{UE}$ = 0.9m may reduce interference and provides better coverage.

## 6. Conclusion

In this paper, we have presented an investigation and analysis on the joint impact of wall and human blockages on uplink performance in a single-tier THz network. The system model have been developed keeping in consideration some of the promising applications like haptic and holographic communications, where the user is presented in an indoor room in the presence of blockages caused by the human body and walls. In the research, we have presented a comparative analysis of 2D and 3D antenna models to provide insight into the impact practical antenna models have on the uplink performance of the THz network. Through this investigation, analytical expressions for various network performance parameters have been observed, which include aggregate interference, MIP, UCP, and ASE. The impact of MIP on TAP density and human blockage density have also been analyzed. The result shows that as the TAP density becomes greater, the MIP increases. Hence, MIP is directly proportional to the $\lambda_{TAP}$. Whereas as the human blockage density becomes greater, the MIP decreases. This is because of interfering signal probability blocked by the human blockage becomes higher. The results suggest that employing the higher antenna gains at TAP and UE enhances the UCP because it creates an impact on the received signal that is higher than the interference. Moreover, as evident from the results, the UCP improves at the higher SINR for PLEs $\left\{\alpha_{TLOS},\alpha_{TNLOS}\right\}$ = {2,4} than $\left\{\alpha_{TLOS},\alpha_{TNLOS}\right\}$ = {3,5}. In the prior studies, an oversimplified 2D antenna model has been employed in which the model accounts only for the horizontal direction, ignoring the impact of interfering links in the vertical direction. For simplicity, the antenna gains of TAP and UE are the same. As a result, the impact of THz communications in the presence of human and wall blockage in an indoor environment under various environmental and network parameters are inaccurate. However, employing a 3D antenna model that accounts for both horizontal and vertical directions provides more realistic results in the same scenarios. The results suggest that UCP by employing lower UE height at desk level ($H_{UE}$ = 0.9m) achieves better coverage at higher SINR. This is because it experiences more blockages caused by human blocker, which ultimately reduces interference from the other users. Moreover, the UCP by employing higher UE height at head level ($H_{UE}$ = 1.3m) performs better coverage at low SINR, as increased UE height improves LOS probability and elevation alignment, resulting into stronger received signal despite of noise. Therefore, the transmission distance in THz communication is limited to only a few meters, it is necessary to employ both the horizontal and vertical heights for more accurate results.

## 7. Appendix A

The LSF for the LOS $Lf_{THz,LOS}\left(x_{iTH}\right)$ can be expressed as,


$$\begin{array}{c} Lf_{THz,LOS}\left(x_{iTH}\right)={\left(\frac{c}{4\pi f_{TAP}}\right)}^{\text{2}}{x_{iTH}}^{-\alpha_{TLOS}}e^{-K\left(f_{TAP}\right)\;x_{iTH}}\;{d_{iT}}^{\alpha_{TLOS}\tau } \end{array}$$ (39)

and the LSF for the NLOS $Lf_{THz,NLOS}\left(x_{iTH}\right)$ can be expressed as,


$$\begin{array}{c} Lf_{THz,NLOS}={\left(\frac{c}{4\pi f_{TAP}}\right)}^{\text{2}}{x_{TH}}^{-\alpha_{TNLOS}}e^{-K\left(f_{TAP}\right)x_{TH}}\;{d_{iT}}^{\alpha_{TNLOS}\tau }\text{\textbackslash{}hspace\{0.33em}\}𝔼\left[{R_{C}}^{\text{2}}\right]\text{\textbackslash{}hspace\{0.33em}\} \end{array}$$ (40)

where the distance between TAP and an interfering UE is expressed as $x_{iTH}$ and the distance of the serving TAP from the interfering UE is represented by $d_{iT}$. The Laplace transform of aggregate Interference of the THz tier is computed using the following expression.


$$\begin{array}{c} {ℒ}_{I_{agg,THz}}\left(s\right)=𝔼\;\left[e^{-s\;I_{agg,THz}}\right] \end{array}$$ (41)

where $s\in \left[s_{TL},s_{TNL}\right]$ are the LOS and NLOS links denoted as,


$$\begin{array}{c} s_{TL}=-\frac{nT\beta \left(x_{TH}\right)\text{\textbackslash{}hspace\{0.33em}}{\gamma }\left(x_{TH}\right)\}P_{UE}G_{i}Lf_{THz,LOS}\left(x_{TH}\right),\text{\textbackslash{}hspace\{0.33em}\}s_{TNL}=-\frac{T}{P_{UE}G_{i}Lf_{THz,NLOS}\left(x_{TH}\right)} \end{array}$$ (42)

where $\gamma \left(x_{TH}\right)$ follows the gamma distribution. By putting equations (34) and (42) in (41), the following equation is obtained.


$$\begin{array}{c} {ℒ}_{I_{agg,THz}}\left(s\right)={𝔼}_{I}\left[exp\left(-s\sum_{x_{iTH}\in \Phi_{I_{T}}}P_{UE}G_{i}S\;f_{THz}\left(x_{iTH}\right)Lf_{THz}\left(x_{iTH}\right)\right)\right] \end{array}$$ (43)

Using the independence of random variables $G_{i}$, $d_{iT}$, $Sf_{THz}$ across $\Phi_{I_{T}}$, can be denoted as,


$$\begin{array}{c} {ℒ}_{I_{agg,THz}}\left(s\right)={𝔼}_{\Phi_{I_{T}}}\left[\prod_{x_{iTH}\in \Phi_{I_{T}}}{𝔼}_{G_{i}Sf_{THz}}exp\left(-sP_{UE}GSf_{THz}\left(x_{iTH}\right)Lf_{THz}\left(x_{iTH}\right)\right)\right] \end{array}$$ (44)

$$\begin{array}{c} {ℒ}_{I_{agg,THz}}\left(s\right)=exp\left(-2\pi \lambda_{TAP}\overset{R_{TH}}{\underset{x_{TH}}{\int }}\left(1-𝔼\left[e^{sP_{UE}GSf_{THz}\left(x_{TH}\right)Lf_{THz}\left(x_{TH}\right)}\right]\right)x_{TH}\text{\textbackslash{}hspace\{0.33em}\}dx_{TH}\right) \end{array}$$ (45)

where $dis_{T}\left(x_{TH}\right)=\lambda_{TAP}\left(1-exp\left(-\pi \lambda_{TAP}{x_{TH}}^{\text{2}}\right)\right)$ is the interference field from serving TAP, which is a non-homogeneous PPP denoted by $\Phi_{I_{T}}$.

## 8. Appendix B

The conditional coverage probability $P_{C,TLOS}\left(T\right)$ when a user connects with the LOS link to TAP with a distance $x_{TH}$ can be stated as,


$$\begin{array}{c} P_{C,TLOS}\left(T\right)=ℒ\left(S\;f_{THz,LOS}\left(x_{TH}\right)>\frac{T\left(I_{agg,THz}+{\sigma^{\text{2}}}_{Noise}\right)}{P_{UE}G_{TAP}G_{UE}Lf_{THz,LOS}\left(x_{TH}\right)}\right)\text{\textbackslash{}hspace\{0.33em}\}=\overset{\infty }{\underset{n=1}{\sum }}{\left(-1\right)}^{n+1}\left(\begin{array}{c} @c@a\left(x_{TH}\right)\\ n \end{array}\right)e^{s_{TL}{\sigma^{\text{2}}}_{Noise}}I_{agg,THz}\left(s_{TL}\right) \end{array}$$ (46)

The conditional coverage probability $P_{C,TNLOS}\left(T\right)$ when a user connects with the NLOS link to TAP at a distance $x_{TH}\text{\textbackslash{}hspace\{0.17em}\}$ can be stated as,


$$\begin{array}{c} P_{C,TNLOS}\left(T\right)=ℒ\left(S\;f_{THz,NLOS}\left(x_{TH}\right)>\frac{T\left(I_{agg,THz}+{\sigma^{\text{2}}}_{Noise}\right)}{P_{UE}G_{TAP}G_{UE}Lf_{THz,NLOS}\left(x_{TH}\right)}\right)\text{\textbackslash{}hspace\{0.33em}\}=e^{s_{TNL}{\sigma^{\text{2}}}_{Noise}}I_{agg,THz}\left(s_{TNL}\right) \end{array}$$ (47)
